# Optimizing learning and motor performance in highly trained youth football players: the role of time interval between synchronous and asynchronous sessions in a blended training model

**DOI:** 10.3389/fspor.2025.1736195

**Published:** 2026-01-06

**Authors:** Aymen Haweni, Amayra Tannoubi, Maha Gasmi, Vasile Emil Ursu, Vlad Adrian Geantă

**Affiliations:** 1 The Higher Institute of Sport and Physical Education (Ksar Saïd), University of Manouba, Manouba, Tunisia; 2 Physical Activity, Sport and Health, Research Unit (UR18JS01), National Observatory of Sport, Tunis, Tunisia; 3 Department of Education, High Institute of Sport, and Physical Education of Gafsa, University of Gafsa, Gafsa, Tunisia; 4 Sports Performance Optimization Research Laboratory (LR09SEP01), National Center for Sports Medicine and Science (CNMSS), Tunis, Tunisia; 5 Research Laboratory (LR23JS01) “Sport Performance, Health & Society”, Higher Institute of Sport and Physical Education of Ksar Saîd, University of Manouba, Tunis, Tunisia; 6 Department of Physical Education and Sport, Faculty of Law and Social Sciences, University “1 Decembrie 1918” of Alba Iulia, Alba Iulia, Romania; 7 Department of Physical Education and Sport, Faculty of Physical Education and Sport, Aurel Vlaicu University of Arad, Arad, Romania

**Keywords:** cognitive performance, football, motor control, neurocognitive integration, perceptual–motor learning, skill acquisition, temporal spacing, working memory

## Abstract

**Background/objectives:**

Effective motor performance in sport depends on the dynamic interaction between cognitive and physiological systems. However, it remains unclear how the temporal spacing between training sessions influences this interaction. This study examined the effects of the Time Interval Between Synchronous and Asynchronous Sessions (TIBSAS) on motor control and working memory in highly trained youth football players.

**Methods:**

Fifty-seven adolescent athletes (12.1 ± 0.9 years) participated in a randomized crossover design involving three interval conditions between training sessions: (1) no delay, (2) 6–12 h delay, and (3) 12–24 h delay. Motor performance was evaluated using a 15 m ball-dribbling test, and cognitive performance was assessed using the Sternberg working memory task. Data were analyzed with one-way repeated-measures ANOVA with Bonferroni correction.

**Results:**

TIBSAS had significant impacts on both motor and cognitive performance. The 12–24 h interval (COND 3) produced considerably quicker dribbling times (3.60 ± 0.16 s) in comparison to COND 1 (3.96 ± 0.21 s, *p* < 0.001) and COND 2 (4.07 ± 0.23 s, *p* < 0.001), exhibiting a substantial effect size (*η*^2^ = 0.50). Cognitive performance exhibited analogous enhancements, with COND 3 indicating significantly quicker reaction times for both one-item (733.74 ± 13.08 ms vs. 777.15 ± 41.91 ms, *p* < 0.001) and three-item memory loads (982.00 ± 40.19 ms vs. 1,022.30 ± 33.10 ms, *p* = 0.005). There were no significant differences in the five-item memory load.

**Conclusion:**

An optimal spacing of 12–24 h between training sessions enhances both motor execution and cognitive processing, suggesting improved integration of perceptual and executive systems in young athletes. These findings highlight the importance of time-dependent consolidation mechanisms in sport performance and offer practical guidance for designing cognitively informed training schedules.

## 1 Introduction

Rapid technological advancements and a growing demand for flexible, remote, and hybrid learning options are driving a major shift in sports coaching (1). Blended learning (BL), which combines synchronous (live, instructor-led) and asynchronous (self-paced, pre-recorded) sessions, has become a popular approach to teach athletes new skills and competencies in this dynamic environment (2–5). For football players, BL offers benefits such as customized learning paths, flexible training schedules, and improved practice time management. This is especially important for young athletes, whose social, athletic, and academic obligations frequently compete for their attention (6).

Beyond its pedagogical implications, blended learning also provides a unique framework to explore the interaction between cognitive and motor processes in sport training. The alternation between synchronous and asynchronous modes inherently engages perception, attention, and memory mechanisms, making it an ideal context for studying how cognitive and perceptual functions shape motor control and performance.

Recent studies indicate that BL can significantly improve motor skills, cognitive understanding, and motivation across various sports disciplines compared to traditional face-to-face instruction (6). For example, in gymnastics, blended learning significantly enhanced technical execution and knowledge retention in secondary school students (7). Similarly, basketball programs employing BL have shown notable improvements in coordination, physical performance, and commitment to training regimens (5).

Although the structural benefits of BL are increasingly recognized, little is known about the optimal timing of sessions, which may critically influence the critical post-practice consolidation phase (8). The Time Interval Between Synchronous and Asynchronous Sessions (TIBSAS) represents an important pedagogical factor that relates to both motor learning theory and cognitive science (9, 10). The testing effect (practice that emphasizes recall enhances memory) and the spacing effect (practice that is distributed improves retention) show that the order and timing of learning activities are very important for strengthening knowledge and skills (11–13). Short intervals may not provide sufficient time for memory stabilization and synaptic consolidation. This may result in proactive interference, wherein the second session disrupts the memory traces of the first (14). In the present study, the asynchronous session directly applies these principles by requiring athletes to actively retrieve, discriminate, and update perceptual-cognitive information without physical execution. Such self-paced tasks emphasize retrieval-based learning, aligning with the testing effect (15), while their placement after the synchronous sessions allows temporal spacing to support consolidation processes central to the spacing effect (16). In football, technical motor skills like dribbling, passing accurately, and controlling the ball with higher-level cognitive skills like working memory, attention management, and fast decision-making. The best TIBSAS is probably one that lets the motor memory be processed and added to offline without too much trouble or damage. Planning the time you have wisely is really important if you wish to learn hard skills (17). Recent studies on motor-cognitive training show that adding cognitive tasks to agility drills makes it easier to learn and remember new skills (18).

Despite theoretical understanding of the significance of temporal spacing, there is insufficient empirical data on TIBSAS in athletic contexts. Most research on blended learning in sports and physical education has contrasted it with traditional methods, often neglecting session duration (4, 5, 19). Research in basketball, gymnastics, and sailing indicates that the scheduling of asynchronous information vs. live instruction might influence motivation, skill acquisition, and retention (4, 5, 7, 20, 21). Individual traits such as chronotype, fatigue, and cognitive stress may influence the efficacy of TIBSAS (22). Even though BL has clear structural benefits and learning theory has well-documented reasons for using time spacing, there isn't enough empirical evidence looking into the best time interval between synchronous and asynchronous sessions (TIBSAS) in sports. Previous research has mostly compared BL to traditional approaches, but the exact temporal parameters that enhance cognitive and motor consolidation are still not known. Thus, this study seeks to explicitly examine the impact of varying TIBSAS intervals on the motor and cognitive performance of youth football players, thereby addressing a critical gap between motor learning theory and practical training implementation.

Based on the principles of distributed practice and motor memory consolidation, we hypothesized that:

H1: A longer TIBSAS (12–24 h) would lead to superior motor performance compared to shorter (6–12 h) or no intervals.

H2: The same 12–24 h TIBSAS would also improve cognitive performance, as evidenced by faster reaction times on a working memory task.

## 2 Materials and methods

### 2.1 Participants

Using G*Power (23), we figured out the required sample size. The alpha level was 0.05, the power was 0.95, and the projected effect size was 0.71 based on a previous study investigating working memory in youth (22), reflecting an expected large effect consistent with the potent impact of cognitive-motor interventions. This resulted in a minimum of 14 participants for each group.

A total of 62 male youth football players from three clubs were screened, with five excluded for not completing the protocol. The final sample consisted of 57 participants (mean age: 12.11 ± 0.92 years; height: 149.25 ± 3.09 cm; body mass: 54.39 ± 1.63 kg). Following a thorough explanation of the testing protocols and experimental conditions, all participants as well as their parents or legal guardians provided written informed consent prior to participation. The study adhered to the Declaration of Helsinki (24) and was approved by the Ethics Committee of the High Institute of Sports and Physical Education of Ksar-Saïd, University of Manouba, Tunisia (Approval reference: 17/2025, dated 17-01-2025), in line with established guidelines in sports medicine and exercise science (25).

### 2.2 Eligibility criteria

The participants were 12–13-year-old, nationally competitive, highly skilled football players (Tier 3) (26). The inclusion criteria were: above-average physical and technical skills, involvement in at least five training sessions per week (8–10 h), and a minimum of 2 years of consistent training, validated by an official medical certificate (27, 28). The Morningness-Eveningness Questionnaire (MEQ; scores 42–58) was used to determine chronotype, which put participants in the intermediate chronotype group. This could affect motor function, vigilance, and reaction time (29).

Exclusion criteria encompassed significant absenteeism, injuries, health concerns impacting performance, or categorization below Tier 3 (26). These criteria made sure that the sample of well-trained youth football players was all the same and could follow the whole protocol.

### 2.3 Research design

Every participant experienced all three experimental conditions: (i) a control condition without TIBSAS, (ii) a 6–12-hour interval, and (iii) a 12–24-hour interval, presented in a counterbalanced order across three consecutive weeks. The within-subject design diminished inter-individual variability and enhanced the sensitivity of statistical comparisons. An independent computer program (http://www.radomized.org) made randomization sequences, which were kept secret until they were needed. Each club went through a 3-week intervention cycle, with one condition provided each week and at least 72 h between conditions to avoid any possible carryover or weariness effects.

### 2.4 Procedures

All assessment were conducted on the club's training grounds following standardized setups. Each participant completed two primary assessments during every testing session, performed in randomized order. (i) The 15-m Ball Dribbling Test (Ball-15m) for technical performance (27), and (ii) the Sternberg Working Memory Task for cognitive performance (30). Participants were instructed to maintain consistent routines, avoid additional physical exertion, and adhere to regular sleep patterns throughout the experiment. Parents monitored adherence and reported any deviations to the research team. Testing sessions were held in controlled environments with a temperature range of 21 °C–23 °C and relative humidity of 45%–55% (31). They were also held at the same time every day (10:00–12:00 A.M.) to account for changes in performance throughout the day (32). To ensure diurnal control and avoid acute fatigue effects, synchronous field sessions for all conditions were always conducted between 10:00 and 12:00. The asynchronous sessions were scheduled according to the interval required by each condition: immediately after the synchronous session (COND 1), between 16:00 and 22:00 (COND 2), or between 22:00 and 10:00 the following day (COND 3). This explicit sequencing ensures that cognitive assessments always occurred after a standardized recovery window. We used a single-blind protocol. Participants did not know what the experiment was about, and the people who judged them did not know which condition they were in. There was a 2-week period of getting used to the test before the real test to reduce the impacts of learning and expectations. During this time, participants practiced all testing methods under the watchful eye of the investigator (A.H.) to make sure they were reliable. This design ensures strong internal validity, allowing performance variances to be securely ascribed to differences in TIBSAS, rather than uncontrolled confounding variables such as fatigue, motivation, or circadian rhythms.

#### 2.4.1 Technical performance—15-m Ball Dribbling Test (Ball-15m)

**Figure 1:**
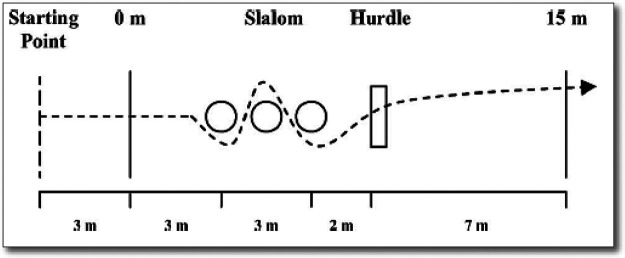
Schematic representation of Ball-15m (27).

This test assessed dribbling speed, ball handling, coordination, and agility. Participants started behind a marked line, dribbled through a 15-m course with a passive slalom, kicked the ball under a hurdle toward one of two small goals, and sprinted to the finish line (Figure 1). Two maximal attempts were recorded, with a 3-min rest between trials. The fastest time was used. Sprinting speed was measured with photocell gates (Timer S4, Alge-Timing, Lustenau, Austria).

The Ball-15m test demonstrates high reliability in youth soccer players (ICC = 0.85–0.95). Standardized procedures, including consistent course setup, instructions, and photocell timing, minimized measurement error (27, 33).

#### 2.4.2 Working memory

Working memory was assessed using the Sternberg Paradigm (30) on a Lenovo ThinkPad T470s laptop. Average session duration was approximately 8 min. Instructions were displayed on-screen before each run, followed by a rehearsal period during which no data was recorded. Each participant completed the test individually in a quiet, dimly lit room, wearing noise-canceling headphones to minimize distractions.

The task had three levels of difficulty, with memory loads of one, three, and five items. In the one-item condition, the number “3” was the target and there were 16 stimuli. The three-item and five-item conditions used letter sets that were randomly generated and had 32 stimuli each. At the beginning of each task, targets were shown, and participants used the right arrow key for targets and the left arrow key for distractions to respond. There was a 1-second gap between each item, and they were all in the middle of the screen. The measured outcomes comprised reaction time (ms) for proper targets and response accuracy (% correct).

#### 2.4.3 Training sessions

The study adopted a blended training approach where each experimental condition comprised of one synchronous (live) session followed by one asynchronous (self-paced) session, separated by the designated TIBSAS.

##### 2.4.3.1 Synchronous session

These were 50-minute, coach-led sessions done on the field. The curriculum was standardized and focused on ball mastery and dribbling under pressure, directly relevant to the Ball-15m evaluation. The head coach of each club, who was trained by the primary investigator (A.H.) on a standardized protocol, led all the sessions to make sure they were all the same. Table 1 shows how the session is set up.

**Table 1:** Training sessions description.

Session component	Synchronous session	Asynchronous session
Objective	Improve dribbling proficiency, controlling the ball, and being quick on your feet when there isn't much pressure.	Enhance decision-making processes, perceptual-cognitive processes, and mental rehearsal.
Content	Warm up for 5 min.	Instruction based on video (5 min).
	Drills include slalom dribbling, 1v1 feints, and turns under pressure.	Cognitive tasks: Watching game video and figuring out which options are available.
	A small-sided game (4v4) that focuses on dribbling.	Mirror drills: copying the footwork patterns seen on the screen.
Duration	50 min	25 min
Tools/Platform	Cones, balls, hurdles, photocell gates (for feedback).	Private YouTube playlist, personal computer/tablet.
Coach Involvement	Direct instruction, feedback, and demonstration by a trained coach.	No direct coach involvement. Instructions were pre-recorded.
Feedback	Immediate, verbal feedback from the coach.	Intrinsic feedback based on video comparison; no external feedback.

##### 2.4.3.2 Asynchronous session

These were 25-minute self-paced sessions performed by participants at home. Only participants could see the content through a private YouTube playlist. The sessions included cognitive-motor exercises that used videos, like viewing and copying certain footwork patterns and responding to visual cues about making decisions. These tasks were meant to improve on working memory and perceptual skills without using a ball. Table 2 gives a full analysis.

**Table 2:** Motor performance (Mujika test) and cognitive reaction times (Sternberg Paradigm) across TIBSAS conditions.

Tests	COND 1	COND 2	COND 3	RM-ANOVA	*Post-hoc* comparisons (*p*-value)				
				*F*	*p*	*η* ^2^	COND 1 vs. 2	COND 1 vs. 3	COND 2 vs. 3
Ball-15m (s)	3.96 ± 0.21	4.07 ± 0.23	3.60 ± 0.16	*F*_(2, 112)_ = 25.10	<0.001	0.497	*p* = 0.210	*p* < 0.001	*p* < 0.001
Sternberg paradigm									
One-item (ms)	777.15 ± 41.91	744.06 ± 22.99	733.74 ± 13.08	*F*_(2, 112)_ = 10.95	<0.001	0.309	*p* = 0.015	*p* < 0.001	*p* = 1.000
Three-item (ms)	1,022.30 ± 33.10	1,020.41 ± 35.48	982.00 ± 40.19	*F*_(1.6, 89.6)_ = 7.15	0.002	0.218	*p* = 1.000	*p* = 0.005	*p* = 0.012
Five-item (ms)	1,241.15 ± 38.02	1,226.76 ± 37.09	1,229.63 ± 61.82	*F*_(2, 112)_ = 0.49	0.613	0.018	*p* = 1.000	*p* = 1.000	*p* = 1.000

TIBSAS, time interval between synchronous and asynchronous sessions. Values are presented as mean ± standard deviation. COND1 = no TIBSAS; COND2 = TIBSAS 6–12 h; COND3 = TIBSAS 12–24 h. *F*, RM-ANOVA *F* statistic; *p* = significance value; *η*^2^, effect size. Data were analyzed using a one-way repeated-measures ANOVA with Bonferroni correction for *post-hoc* comparisons. For the three-item Sternberg task, the Greenhouse-Geisser correction was applied due to a violation of sphericity (*ε* = 0.80), hence the reported degrees of freedom are adjusted.

##### 2.4.3.3 Adherence and fidelity

To make sure that participants finished the asynchronous sessions, parents kept an eye on them, and digital analytics (video viewership data from YouTube) were used. The mean actual interval for the 6–12 h condition was 9.2 ± 1.8 h.

### 2.5 Statistical analyses

**Figure 2:**
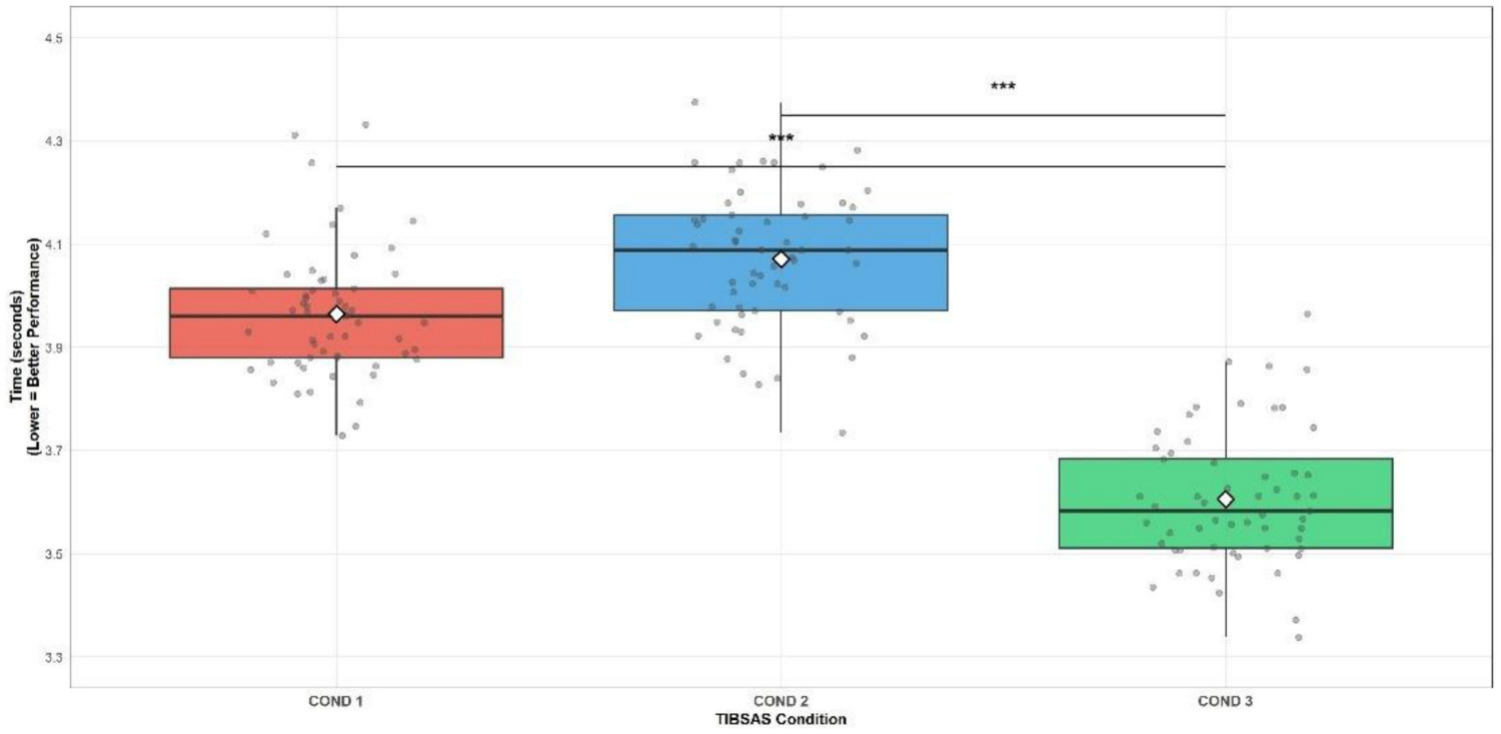
The Ball-15m across TIBSAS conditions.

**Figure 3:**
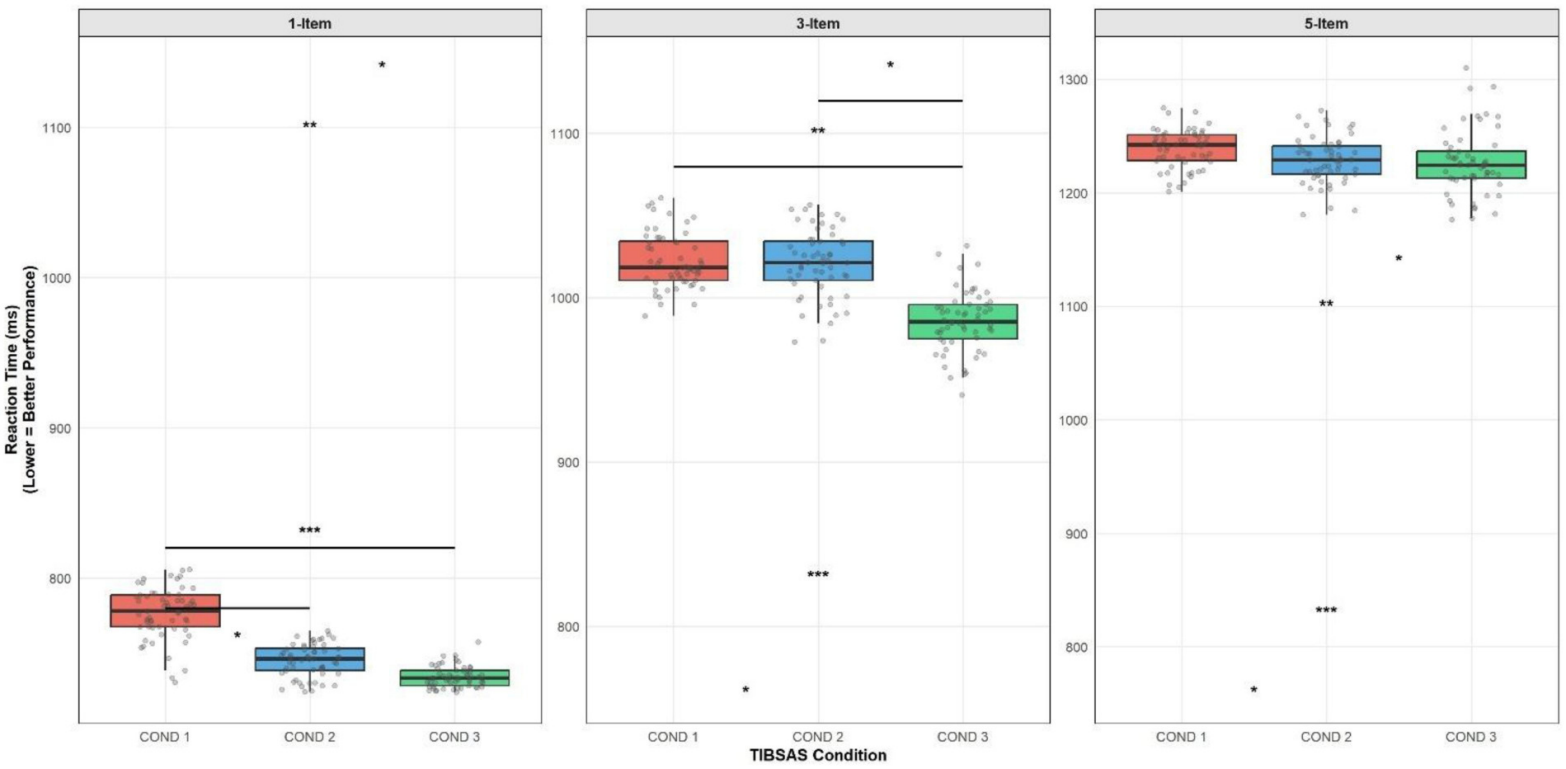
Reaction time (ms) at the cognitive performance across TIBSAS conditions.

**Figure 4:**
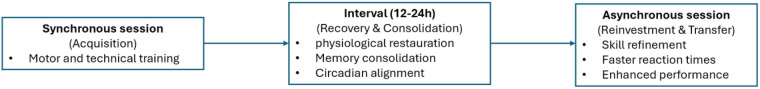
TIBSAS mechanism and its effects on motor and cognitive performance.

Data are presented as means ± SD (Table 1) and standard errors ± SE (Figures 1–4). The Shapiro–Wilk test was used to check for normality, and it showed that the distribution was normal. One-way repeated measure ANOVAs were used to examine technical performance and working memory outcomes across the three experimental conditions (COND1, COND2, and COND3). *Post-hoc* multiple comparisons were performed following significant main effects. Effect sizes were calculated using eta-squared (*η*^2^) and interpreted according to Cohen's criteria (small: *η*^2^ ≥ 0.01; medium: *η*^2^ ≥ 0.06; large: *η*^2^ ≥ 0.14) (34). Analyses were conducted using IBM SPSS Statistics (24.0) for Windows. The RStudio version 2025.05.0 for Windows (61) was used to produce the figures. The *ggplot2*, *ggtext*, and *showtext* packages were used (35–37).

The significance level for all analyses was *p* < 0.05.

## 3 Results

The effects of TIBSAS on motor and cognitive performance are summarized in Table 2.

### 3.1 Technical performance

The Ball-15m revealed significant main effects of TIBSAS conditions, *F*_(2, 112)_ = 25.10, *p* < .001, *η*^2^ = .497, indicating a large effect size. *Post-hoc* test with Bonferroni correction showed that performance in COND 3 (TIBSAS 12–24 h; 3.60 ± 0.16 s) was significantly faster than both COND 1 (no TIBSAS 3.96 ± 0.21 s, *p* < .001) and COND 2 (TIBSAS 6–12 h; 4.07 ± 0.23 s, *p* < .001). There was no statistically significant difference between COND 1 and COND 2 (*p* = .210) (Figure 2).

### 3.2 Cognitive reaction times (Sternberg Paradigm)

For the 1-item level, a repeated-measures ANOVA revealed a significant main effect of condition, *F*_(2, 112)_ = 10.95, *p* < .001, *η*^2^ = .309. *Post-hoc* analysis indicated that reaction times in COND 3 (733.74 ± 13.08 ms) were significantly faster than in COND 1 (777.15 ± 41.91 ms, *p* < .001). The difference between COND 2 (744.06 ± 22.99 ms) and COND 1 also reached significance (*p* = .015), while COND 2 and COND 3 did not differ significantly (*p* = 1.000) (see Figure 3).

The assumption of sphericity was not met at the three-item level; hence the Greenhouse-Geisser adjustment was used (*ε* = 0.80). The analysis revealed a significant main effect, *F*_(1.6, 89.6)_ = 7.15, *p* = .002, *η*^2^ = .218. *Post-hoc* testing indicated that COND 3 (982.00 ± 40.19 ms) resulted in considerably quicker reaction times in comparison to both COND 1 (1,022.30 ± 33.10 ms, *p* = .005) and COND 2 (1,020.41 ± 35.48 ms, *p* = .012). COND 1 and COND 2 were the same (*p* = 1.000) (See Figure 3).

For the five-item memory load, there was no significant main effect of TIBSAS condition, *F*_(2, 112)_ = 0.49, *p* = .613, *η*^2^ = .018, and all *post-hoc* comparisons were non-significant (*p* = 1.000 for all) (see Figure 3).

## 4 Discussion

The present study aimed to demonstrates that the Time Interval Between Synchronous and Asynchronous Sessions (TIBSAS) affects both technical motor performance and cognitive reaction times, with the longest interval evaluated (COND 3: 12–24 h) producing the most advantageous outcomes for Ball-15m performance and 1- and 3-item working-memory loads as confirmed by the repeated-measures ANOVA results with no effect emerged at the highest 5-item load. However, a nuanced analysis revealed that these advantages were not universal across all performance domains and were contingent on specific task demands.

Our main finding is that a TIBSAS of 12–24 h provide an optimal distributed practice schedule for highly trained youth football players in terms of acute technical performance, as evidenced by a large effect size (*η*^2^ = 0.50) and improving cognitive performance under low-to-moderate working-memory demands (1- and 3-item loads). The large effect size observed for motor performance underscores the substantial practical significance of this interval. This extended interval likely strengthens motor pathways, reduces inter-session interference, and enhances skill learning, consistent with previous evidence (14, 38–40). In contrast, the effects of 6–12 h interval (COND 2) were mixed: it produced a significant improvement in the simplest working-memory condition (1-item load), while showing no benefits or impairment for motor performance or higher cognitive loads. Therefore, COND 2 cannot be interpreted as detrimental but rather as yielding partial, task-dependent effects (41–44). Our findings indicate that COND 2 resulted in markedly slower dribbling speeds in comparison to COND 3, with no significant enhancement shown over COND 1 for Ball-15m performance and for 3- and 5-item reaction times, and only a modest improvement over COND 1 at the 1-item load. Conversely, extended intervals facilitate long-term potentiation (LTP), synaptic plasticity, and consolidation, hence enhancing the stabilization of motor patterns and the retention of skills over the long term (45–47). These longer intervals also help new motor representations form, lower proactive interference, and improve physiological and neurological adaptation by keeping training load stable (48).

Longer TIBSAS breaks also boost motivation, lower mental and physical exhaustion, and make people more interested in and committed to regular training (49). The cognitive results show an important condition: the benefits of TIBSAS were clearer when working memory loads were modest (1-item and 3-item conditions), but there were no significant effects when cognitive demands were high (5-item condition). This trend clearly shows that the advantages of temporal separation are limited by cognitive load and do not apply to tasks that use the most working memory resources. While shorter intervals (COND 1: no TIBSAS; COND 2: 6–12 h) may result in more interference and lower performance outcomes on learning assessments during evaluations, longer intervals will typically yield the following benefits of distributed practice: neuromuscular and metabolic recovery in active recovery, consolidation of motor patterns, improved learning acquisition, retention, and transfer, and an extended duration (12–24 h; COND 3): reinstatement, motivation, and interference limits (50–52).

The study's results showed that practice sessions that were spaced out correctly, especially when they were held 12–24 h apart from synchronous or asynchronous sessions (COND 3, TIBSAS). Notably, these advantages did not extend to the highest (5-item) working memory load. This absence of improvement at the highest load represents an important boundary condition for the spacing effect in sport-specific cognition (53). It suggests that temporal spacing may primarily benefit processes supported by moderate working-memory engagement, while tasks exceeding cognitive capacity limits may not profit from distributed practice due to resources saturation in working memory (54). This null findings suggest boundary condition for the benefits of temporal spacing, indicating that its positive effects may attenuate when cognitive demands exceed a certain threshold, potentially overwhelming the system resources. Integrating these findings with motor outcomes, the results support a shared consolidation mechanism whereby temporal spacing facilitates both prefrontal executive recovery and motor-cortical stabilization (45). Longer intervals may allow cooperative reactivation across prefrontal-motor networks, enhancing both perceptual discrimination and movement execution (55).

Our results are also in line with the literature on motor learning, which examines how memory consolidation and distributed practice contribute to the development and stabilization of motor representations (56). The partial confirmation of our hypotheses through appropriate within-subjects analyses—showing clear benefits of the 12–24 interval for Ball-15m and 1- and 3-item reaction times, but not for the 5-item load—supports these theoretical connections with also highlighting boundary conditions related to cognitive load. Additionally, recent research indicates that spaced practice sessions promote long-term motor skill acquisition and transfer by reducing inter-session interference (14, 57, 58).

Circadian factors pertaining to the Time Interval Between Synchronous and Asynchronous Sessions (TIBSAS) also have an impact on performance. In fact, longer TIBSAS periods, which take place asynchronously within a physiologically optimal compartment during their peak concentration and reactivity, help performers with a moderate chronotype avoid some of the reduced periods of alertness (59). Furthermore, it has been discovered that appropriately spaced practice, indicated by a longer TIBSAS, improves long-term skill acquisition while reducing intersession interference (60).

Importantly, no differences were found at the highest (5-item) memory load, indicating that the influence of TIBSAS may be restricted to low-to-moderate cognitive load levels. This pattern is empirically supported by our findings showing that COND 3 outperformed COND 1 and COND 2 on Ball-15m performance and 1- and 3-item reaction times, whereas no condition differences were detected at the 5-item load. This perspective extends current theories of distributed practice by emphasizing the neurocognitive foundations of motor learning and performance.

### 4.1 Limitations

This study has several limitations. The statistical method employed, specifically repeated-measures ANOVA, effectively addressed within-subject design. Nevertheless, the study design and data collection timeline precluded a reliable examination of potential relationships between cognitive and motor outcomes; outcomes were gathered at varying intervals and with unequal repetitions, precluding a suitable correlation analysis. Furthermore, the sample was only comprised of highly trained teenage football players with an intermediate chronotype, hence constraining generalizability to adults, amateurs, or those with extreme chronotypes. The intervention duration was quite short, constraining conclusions on the longevity of skill development after extended practice. The emphasis was on individual performance metrics rather than team collaboration or group strategies. The regulated field setting may differ from genuine training situations where social, motivational, and environmental factors influence learning. Finally, only two TIBSAS ranges (6–12 and 12–24 h) were examined, indicating the possibility of other unexamined time periods. The study provides valuable insights on optimizing TIBSAS for enhancing evidence-based training regimens, despite its limitations.

### 4.2 Practical implications

Scheduling synchronous and asynchronous sessions 12–24 h apart can improve both working memory and motor skills. This method is in line with the principles of distributed practice and motor learning, which improve cognitive processing, skill retention, and the integration of neuromotor and executive functions. Coaches can also use TIBSAS-based tactics in blended training models to keep people motivated, lower cognitive overload, and make learning more efficient. Changing the length of intervals based on things like chronotype, exhaustion, and training load may help you learn skills and perform better. Evidence-based TIBSAS scheduling can make young football development programs work better and last longer.

## 5 Conclusions

Optimizing the Time Interval Between Synchronous and Asynchronous Sessions (TIBSAS) at 12–24 h consistently benefits both acute motor performance (Ball-15m) and cognitive performance under low-to-moderate working memory loads (1- and 3-item conditions) in highly trained youth football players, while showing no detectable effect at the highest (5-item) load. This period facilitates physiological recuperation, neurocognitive consolidation, and the efficient distribution of cognitive resources, hence potentially enhancing immediate performance, with long-term effects remaining to be verified. In addition to its practical significance, these findings underscore the temporal dynamics governing the interaction between cognitive and perceptual processes that facilitate motor control and learning. They also propose that time-dependent cognitive-motor connection may constitute a fundamental mechanism facilitating effective skill acquisition in sports. These results provide practical, evidence-based guidance for structuring blended or distributed training models and open new directions for research into the neurocognitive optimization of sport performance.

## Data Availability

The raw data supporting the conclusions of this article will be made available by the authors, without undue reservation.
